# A Novel Attempt to Standardize Results of CFD Simulations Basing on Spatial Configuration of Aortic Stent-Grafts

**DOI:** 10.1371/journal.pone.0153332

**Published:** 2016-04-13

**Authors:** Andrzej Polanczyk, Marek Podyma, Lukasz Trebinski, Jaroslaw Chrzastek, Ireneusz Zbicinski, Ludomir Stefanczyk

**Affiliations:** 1 Department of Heat and Mass Transfer, Faculty of Process and Environmental Engineering, Lodz University of Technology, Lodz, Poland; 2 Department of Radiology and Diagnostic Imaging, Medical University of Lodz, Lodz, Poland; Technion - Israel Institute of Technology, ISRAEL

## Abstract

Currently, studies connected with Computational Fluid Dynamic (CFD) techniques focus on assessing hemodynamic of blood flow in vessels in different conditions e.g. after stent-graft’s placement. The paper propose a novel method of standardization of results obtained from calculations of stent-grafts' “pushing forces” (cumulative WSS—Wall Shear Stress), and describes its usefulness in diagnostic process. AngioCT data from 27 patients were used to reconstruct 3D geometries of stent-grafts which next were used to create respective reference cylinders. We made an assumption that both the side surface and the height of a stent-graft and a reference cylinder were equal. The proposed algorithm in conjunction with a stent-graft “pushing forces” on an implant wall, allowed us to determine which spatial configuration of a stent-graft predispose to the higher risk of its migration. For stent-grafts close to cylindrical shape (shape factor *φ* close to 1) WSS value was about 267Pa, while for stent-grafts different from cylindrical shape (*φ* close to 2) WSS value was about 635Pa. It was also noticed that deformation in the stent-graft’s bifurcation part impaired blood flow hemodynamic. Concluding the proposed algorithm of standardization proved its usefulness in estimating the WSS values that may be useful in diagnostic process. Angular bends or tortuosity in bifurcations of an aortic implant should be considered in further studies of estimation of the risk of implantation failure.

## Introduction

First surgical operations with the use of endovascular prostheses to treat aneurysms were made in the 90th of the twentieth century [1–4]. An endovascular prosthesis implantation is proceeded by an estimation of the size and spatial configuration of an aneurysm, as well as selection of a proper type of a stent-graft [5]. Depending on their construction the endovascular prostheses are divided into one-module or multi-modules. In the case of one-module configuration a stent-graft is inserted as one part into the diseased section of the aorta and then expanded. The multi-modules configuration, like in bifurcations, allows placing elements of endovascular implants partially and then to combine them to create one section [6]. Therefore, treatment of Abdominal Aortic Aneurysm (AAA) with endovascular prostheses, especially multi-model configurations, might be associated with an occurrence of complications such as endoleaks in endovascular prostheses, angular bends, migration of prostheses, or appearance of thrombus [7–10].

The use of numerical methods in solving problems related to blood flow in vessels is widely described in the literature [11–15]. Currently, many studies connected with Computational Fluid Dynamic (CFD) techniques focus on assessing hemodynamic of blood flow in vessels after stent-graft’s placement [16], and on a spatial configuration of endovascular implants [17, 18]. However, there are limited premises which enable to modify the stent-grafts’ construction in order to improve its durability and stability [19]. Also systemic conditions make a comparison of homogeneous groups of operated patients a complicated task. Hence, it seems promising to use computer simulations with further verification in clinical observations. However even then the comparison of gathered results and their implementation to other patients is difficult [19].

Therefore, the aim of this study was to use CFD techniques to estimate how stent-graft’s geometry impact the Wall Shear Stress (WSS) value after stent-grafting. We proposed a novel algorithm for standardization of prostheses “pushing forces” (cumulative WSS) and an new attempt to assess its justness and usefulness in the clinical practice.

## Material and Methods

### Study groups

AngioCT data (GE Light-Speed 64 VCT; GE Healthcare, Fairfield, CT, USA) were collected from 27 patients aged 55–78 years who underwent treatment in the Barlicki Hospital No. 2 in Lodz (Poland) between 2007–2012. All participants gave written informed consent to the study. The collected data were from three different types of bifurcated stent-grafts: 16 patients were implanted with Zenith made by COOK (Cook Medical, USA), 8 patients were implanted with Endurant made by Medtronic (Medtronic, USA) and 3 patients were implanted with Excluder made by Gore (Gore, USA). The study was approved by the Local Ethic Committee on Medical University of Lodz (RNN/126/07/KE).

### Preparation of 3D geometries

**Fig 1:**
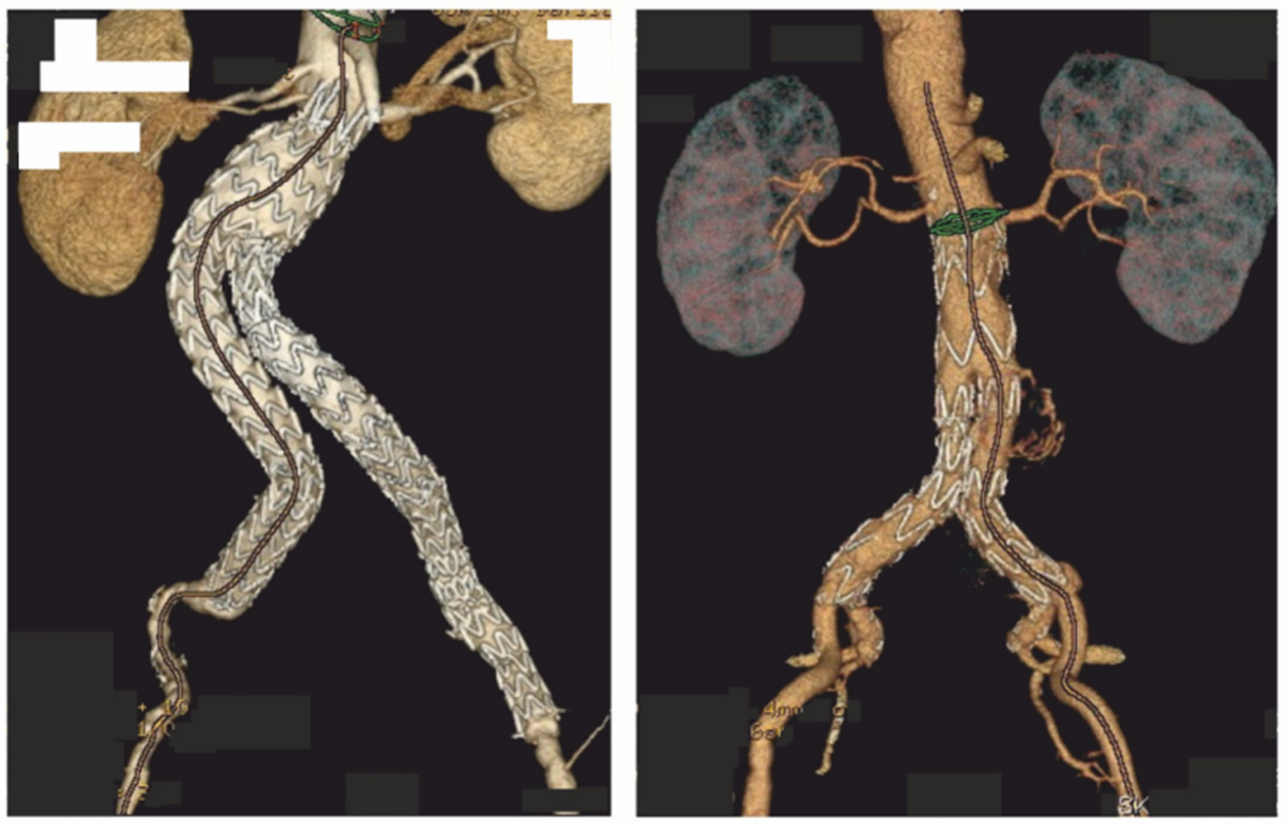
An example for reconstructed spatial configuration of analyzed stent-grafts.

AngioCT data were collected after intravenous contrast injection and were used to reconstruct three-dimensional geometries of bifurcated stent-grafts implanted in the abdominal aortic aneurysm section from the level of the crown of the implant below the iliac branches. A 3DDoctor software (Able Software Corp., Lexington, MA, USA) was used to reconstruct 3D geometries (Fig 1).

### Computer simulations

First, the pre-processor GAMBIT 2.2.30 software (ANSYS Inc., Canonsburg, PA, USA) was used to generate and discretize geometries of a stent-graft. Each of the analyzed geometry was described by a numerical grid composed of 200,000 to 450,000 tetrahedral elements. Numerical calculations of blood flow in the analyzed domains were carried out with the use of ANSYS FLUENT.12.1 software (ANSYS, USA) [20] and restricted by three boundary conditions, velocity-inlet ($\vec{v}$ (x,y,z)), at the outlets from the domain the boundary condition p = const was used and rigid wall (fluid-solid interface, the boundary condition v = 0 was used). Following Johnston et al. blood was treated as non-Newtonian liquid [21]. Blood density had a set value which enabled us to treat it as incompressible liquid (ρ = const) hence, continuity and Navier-Stokes equations were formulated as (Eq 1) and (Eq 2) [22].

∇⋅u=0 (1)

$$\rho \cdot \left(\frac{\partial u}{\partial t}+u\cdot \nabla u\right)=F-\nabla p+\mu \cdot \Delta u$$ (2)

In our study we used the Quemada’s equation to prepare 3D model of blood flow as it was previously described [20] (Eq 3). Reynolds number range was between 1,400 ÷ 1,500.

$$\eta =\eta_{p}\cdot {\left(1-\frac{K\cdot Htc}{2}\right)}^{-2}$$ (3)

where:

ηp—plasma viscosity, [Pa s]

K—inner viscosity of erythrocyte (Eq 4), [–]

Htc—hematocrit, [–]

where:

$$K=\frac{k_{0}+k_{\infty }\cdot {\left(\frac{\gamma }{\gamma^{c}}\right)}^{\frac{1}{2}}}{1+{\left(\frac{\gamma }{\gamma^{c}}\right)}^{\frac{1}{2}}}$$ (4)

where:

k0, k∞—parameters which describes blood character, [–]

γ—shear rate value, [s-1]

γc—critical shear rate value, [s-1]

**Fig 2:**
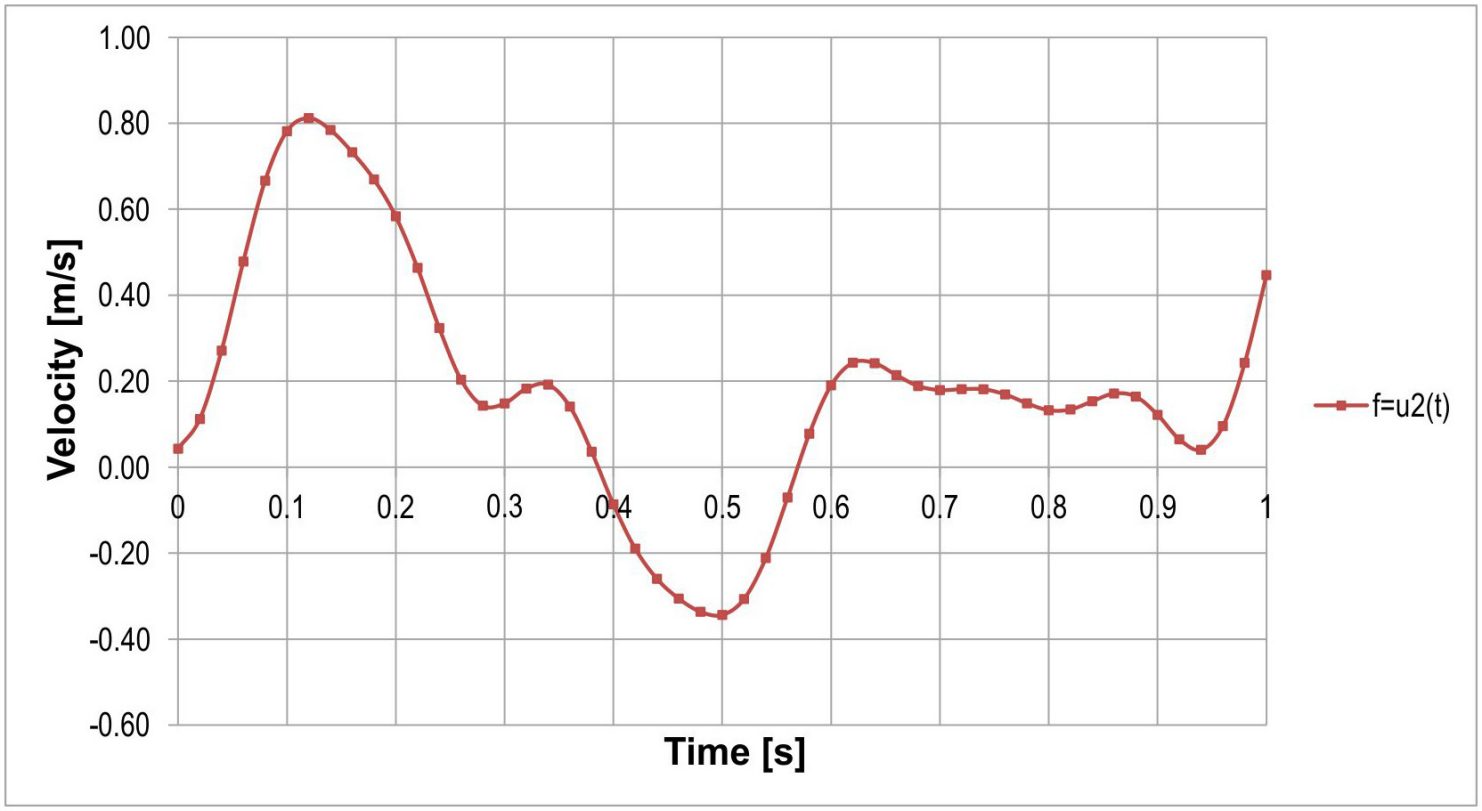
Reference blood velocity profile from USG-Doppler used as a inlet boundary condition for analyzed stent-grafts.

To set the inlet conditions at the inlet of analyzed calculation domains we used the following steps. First, we recorded 27 USG-Doppler (GE Vivid 7, GE Healthcare, USA) examinations from 27 patients. Next, we analyzed the blood velocity profiles (a single flow velocity as a function of time for each patient) paying special attention to the shape of it, especially the appearance of maximum and minimum peaks. We excluded velocity profiles that where too sharp or too flat because that could cause false results giving us extremely high or low (even close to zero) WSS values. Afterwards, we were left with one velocity profile (Fig 2) that meet our expectations and we used it as the "reference blood velocity profile". Hence, were able to compare hemodynamic parameters for all analyzed stent-grafts with the same initial conditions. Furthermore, we set the same initial conditions for all analyzed cases because we investigated the risk of migration by calculating WSS forces acting on stent-graft's wall.

**Fig 3:**
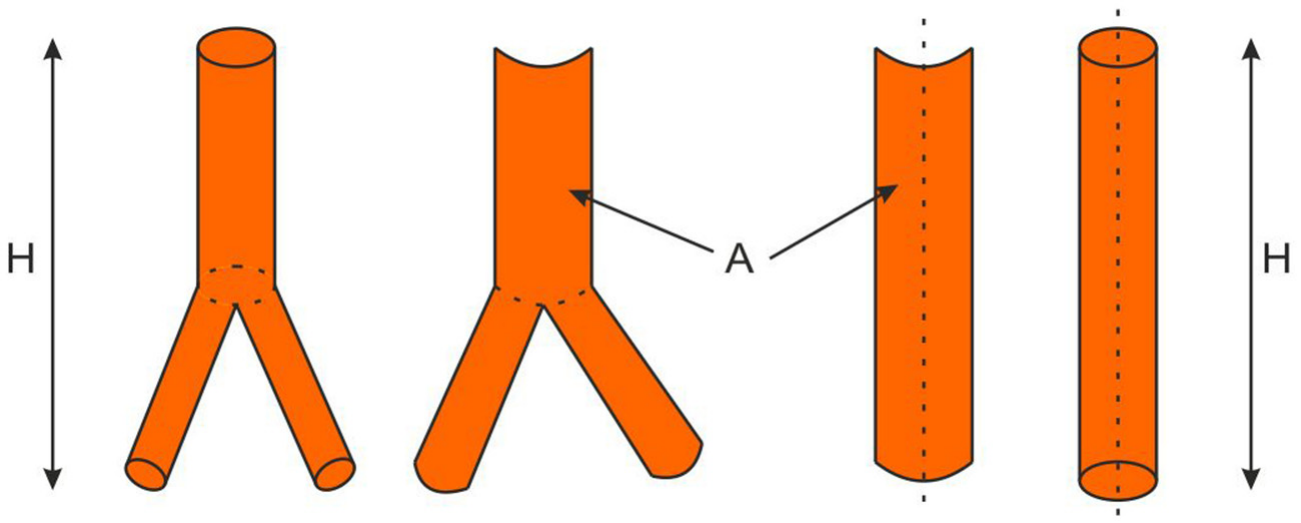
A scheme of reference cylinder for each stent-graft calculation procedure.

During CFD calculations the total WSS value from the implant’s surface for each of analyzed cases was calculated and acquired. In order to standardize the results of the WSS values each shape of a stent-graft was compared to the corresponding reference cylinder, chosen as an ideal geometry with the lowest possible drag coefficient. On the basis of implants geometries, reference cylinders dimensions were calculated. Each cylinder height was assumed to be equal to the related implant height, while cylinder diameter was calculated from stent-graft’s side surface (Eq 5) (Fig 3).

$$A_{R}=\pi \cdot d\cdot h\to d=\frac{A_{R}}{\pi \cdot d}$$ (5)

where:

*A*_*R*_—real side surface of a stent-graft, [m^2^];

*d*—diameter of a reference cylinder, [m];

*h*—height of a reference cylinder and analyzed stent-graft, [m].

Different spatial configuration of analyzed stent-grafts indicated different reference cylinder dimensions. Each stent-graft volume was calculated with the use of the reconstructed 3D geometry, while volume of the reference cylinder (Eq 6) was calculated from the basic cylinder dimensions (the height and the diameter) and the stent-graft’s side surface.

$$V_{w}=\frac{\pi \cdot d^{2}}{4}\to V_{w}=\frac{{A_{R}}^{2}}{\pi \cdot h}$$ (6)

where:

*V*_*w*_—volume of a reference cylinder, [m^3^];

*d*—diameter of a reference cylinder, [m];

*h*—height of a reference cylinder and analyzed stent-graft, [m].

*A*_*R*_—real side surface of a stent-graft, [m^2^];

Finally, we estimated shape factor *φ* (Eq 7) for different spatial configuration of the stent-grafts’ as a relation of the reference cylinder volume determined from the stent-graft’s side surface to the real stent-graft’s volume.

$$\varphi =\frac{V_{w}}{V_{CFD}}$$ (7)

where:

*φ*—shape factor, [–];

*V*_*w*_—volume of reference cylinder, [m^3^];

*V*_*CFD*_—real volume of a stent-graft, [m^3^].

Analysis of shape factors (Eq 7) enabled estimation of differences in blood hemodynamic between optimal configuration of a stent-graft (cylinder shape) for each of the analyzed cases. With this assumption and CFD technique we were able to determine the relation between spatial configurations of the implants and the WSS values during whole cardiac cycle. Instantaneous values of WSS were calculated (frequency = 0.01 sec) as an integral of local shear force values over implant surface in each cardiac cycle interval (Eq 8). Geometries of stent-grafts' closer in diameters to cylindrical tubes indicated higher WSS value.

$$WSS(t)=\int_{A}FdA$$ (8)

where:

*WSS*—shear stress on stent-graft wall, [Pa];

*F*—force acting on a side surface of a stent-graft, [N];

*A*—side surface of a stent-graft, [m^2^].

The total WSS value in whole cardiac cycle, representing total drag force acting on stent-graft’s wall, was calculated as time integral of the instantaneous WSS values (Eq 9).

$$WSS\;tot\sum \left(\frac{WSS(t)}{n}\right)=\frac{1}{n}\cdot \sum WSS(t)=\sum WSS(t)\Delta t$$ (9)

where:

*WSS tot*—shear stress on stent-graft wall, [Pa];

*Δt*—time step, [s]

*n*—number of time steps, [–]

### Statistical analysis

Statistical analysis was performed using GraphPad Prism Version 5.01 (GraphPad Software; San Diego, California, USA). Values were presented as mean±SEM. Correlations were evaluated with the Spearman rank correlation test or Pearson test. Data were considered as significantly different when p<0.05 unless otherwise noted.

## Results

### Wall shear stress values

Analysis of 27 geometries of aortic implants enabled estimation of the influence of spatial configuration and blood hemodynamic on WSS values. Table 1 presents dimensions shape factors and the WSS values of the analyzed stent-grafts. It was noticed that an increase in the shape factor caused an increase in the WSS values.

**Table 1:** The shape factors and WSS values for the analyzed stent-grafts.

No.	Length of supplying distance (length of a stent-graft) [m]	Side surface of a stent-graft [m^2^]	Shape factor [–]	WSS [Pa]
P_1	0.25	0.0245	1.66	407.48
P_2	0.25	0.0254	1.91	393.26
P_3	0.15	0.0119	1.31	435.65
P_4	0.15	0.0114	1.57	546.76
P_5	0.49	0.0538	1.81	720.10
P_6	0.17	0.0171	1.41	268.03
P_7	0.17	0.0179	1.70	314.56
P_8	0.303	0.0306	1.49	337.19
P_9	0.303	0.0323	1.81	406.34
P_10	0.283	0.0254	1.56	314.87
P_11	0.283	0.0193	1.66	353.15
P_12	0.194	0.0188	1.66	590.97
P_13	0.194	0.0199	2.00	635.79
P_14	0.17	0.0130	1.60	352.86
P_15	0.17	0.0140	1.74	389.79
P_16	0.202	0.0224	1.53	345.93
P_17	0.202	0.0238	1.93	414.38
P_18	0.166	0.0162	1.38	310.92
P_19	0.166	0.0162	1.56	364.27
P_20	0.21	0.0190	1.16	267.34
P_21	0.21	0.0181	1.34	391.64
P_22	0.2	0.0199	1.42	446.28
P_23	0.2	0.0216	1.92	515.68
P_24	0.145	0.0128	1.30	348.95
P_25	0.145	0.0135	1.67	403.66
P_26	0.21	0.0120	1.37	698.60
P_27	0.21	0.0117	1.57	824.22

**Fig 4:**
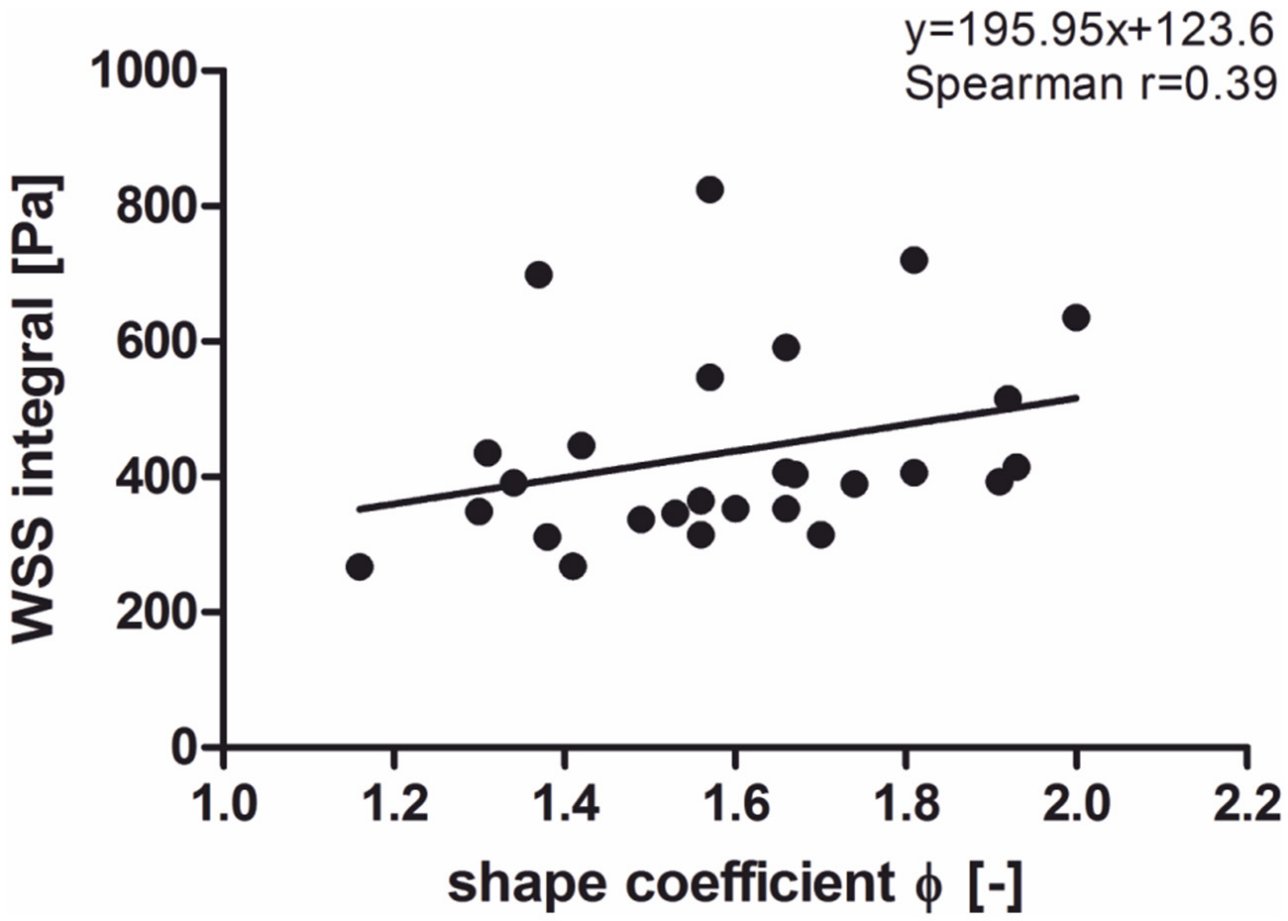
The WSS values in a function of shape factor *φ* for analyzed stent-grafts. Correlation was calculated with Spearman non-parametric test.

Fig 4 presents a relation between the WSS values in a function of stent-graft’s shape factor *φ*. (positive correlation, Spearman r = 0.39). Average value of shape factor for analyzed stent-grafts was *φ* = 1.59 and the average WSS was about 400 ± 28 Pa. For the cases where a shape of a stent-graft was close to a shape of its reference cylindrical tube an average WSS value was about 267 ± 28 Pa (*φ* = 1.15). However when a shape of a stent-graft was lower to a shape of its reference cylindrical tube higher values of WSS (about 635 ± 28 Pa for *φ* = 2.00) were observed.

### Shape factor

**Fig 5:**
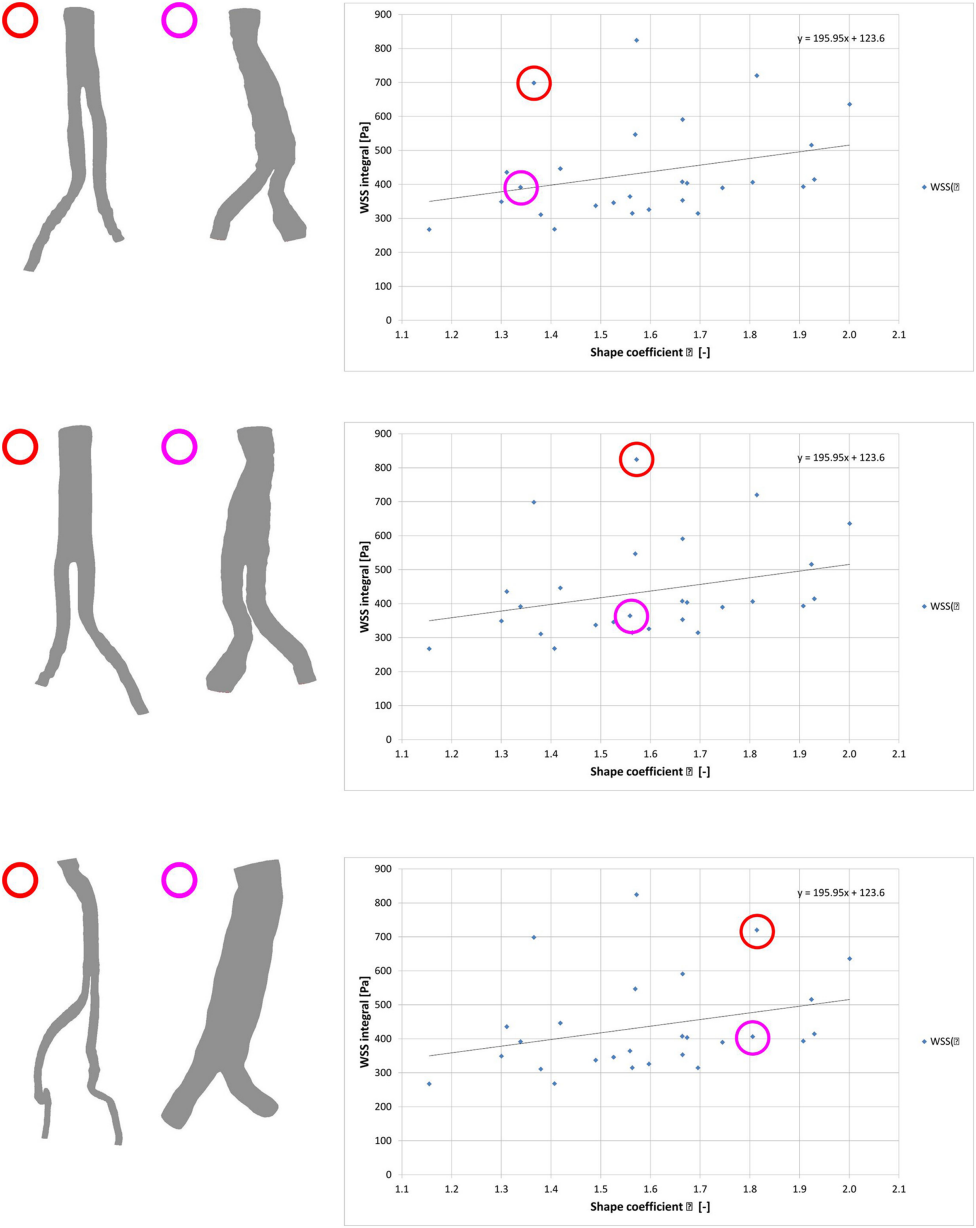
Spatial configuration of stent-grafts that are not on the regression line. With red colour stent-grafts that are not on the regression line. With violet colour corresponding by shape factor, stent-grafts close to the regression line.

Fig 5 presents spatial configurations (shape factors) for all stent-grafts. It was noticed that three geometries markedly differed from a regression’s line (marked in red colour circles in Fig 5). In order to determine why these points outlined we compared them with other stent-grafts with similar shape factor (marked in violet colour circles in Fig 5). We noticed that for the stent-graft with shape factor *φ* = 1.37 the WSS value was about 699 Pa. According to Fig 5 the WSS value should not exceed 400 Pa for stent grafts with similar shape factor.

The two other stent-grafts standing-off the regression line (Fig 5) had shape factor about *φ* = 1.57 and *φ* = 1.81 and the WSS values about 824 Pa and 720 Pa, respectively. Further analysis indicated that those stent-grafts outliers had angular bends and tortuosity in the bifurcation part (Fig 5). Angular bands were calculated as a 180° equal to straight branch and 0° as a entirely bend branch. The highest value of angular bend for the cases on the regression line was about 135°, while for the cases standing-off the regression line was about 87°. Moreover, an average tortuosity (calculated as a relation of distance between parallel planes—inlet/outlet in the iliac part of a stent-graft to distance calculated in axis of iliac part of a stent-graft) for cases standing-off the regression line was about 0.65. Also, for the stent-grafts on the regression line tortuosity was about 0.85 (Table 2).

**Table 2:** Geometrical parameters (angular bends and tortuosity) for the selected stent-grafts. A_L_—angular bend in left branch of a stent-graft, A_R_—angular bend in the right branch of a stent-graft, T_L_—tortuosity of a left branch of a stent-graft, T_R_—tortuosity of a right branch of a stent-graft.

Parameters	P_26	P_21	P_27	P_19	P_5	P_9
Angular bend						
A_L_ [°]	142.4	153.5	140.1	152.9	87.42	134.54
A_R_ [°]	170.2	156.1	169.1	157.9	125.01	143.62
A_L_/A_R_	0.84	0.98	0.83	0.97	0.70	0.94
Tortuosity						
T_L_	0.49	0.86	0.65	0.82	0.58	0.77
T_R_	0.72	0.92	0.70	0.83	0.73	0.90
T_L_/T_R_	0.68	0.93	0.93	0.99	0.79	0.86

Hence, our analysis showed that deformation in bifurcation part of a stent-graft may additionally disturb blood hemodynamic which lead to an increase in the WSS values and, as a result higher risk of stent-graft migration.

## Discussion

The paper presents a novel approach to standardize the results of computer simulations basing on spatial configuration of aortic stent-grafts. We focused on the analysis of the relation between the shape factor and the WSS value on the wall of prosthesis to propose it as a tool for prediction of implant’s migration risk. We proposed an algorithm of standardization useful in estimating how stent-graft’s spatial configuration may disturb blood hemodynamic leading to an increase in WSS values.

However, we are aware that WSS is not the only factor which might induce implant’s migration risk. Clinical observations showed that stent-grafts implanted in aneurysms with short neck, wide sack and widened iliac arteries are more prone to migration contrary to the stent-grafts that are anchored in long neck and normal iliac arteries [23, 24]. Moreover, there is a significant influence of stent-graft’s construction. For instance stent-grafts that are not leaning on the bifurcation of aorta and the ones that are composed of several elements are more prone to migration [25, 26].

The last decade is characterized by a dynamic development of endovascular techniques used in AAA therapy. Nowadays, stent-grafts are a known method of aneurysm therapy and its construction is still improving [27, 28]. It is difficult, however, to obtain relevant arguments without putting patients at risk of innovations [29]. There are no reliable premises which would enable to modify the stent-grafts’ construction in order to improve, both durability and stability (long term observations are here necessary) [28]. The number of independent systemic conditions which have an effect on patient’s survival, implant patency and which hinder inference backed up by statistical evidence, induce a complicated task to obtain a homogeneous group for investigations [27, 29]. Hence, there is a necessity to formulate hypotheses on the basis of computer simulations and their subsequent verification in clinical conditions [30].

In our study we used CFD techniques to analyse different clinical cases to present a link between spatial configuration of the stent-grafts and WSS. We also analyzed the impact of the inlet and outlet diameters of the stent-graft and their orientation in space. It was observed that when stent-grafts were more twisted, the relevant forces were higher and the risk of implant’s migration increased. We believe it is a novel approach as previous works were focused on the analysis of implant diameters [31]. Also, supporting our study there are reports on the analysis of the impact of the angle of the iliac part of the stent-grafts [32].

A comparison of different implant geometries enabled us to estimate the effect of changes in flow conditions on the value of stresses formed in the region of proximal fixation. It was recently described that changes in flow conditions influence stent-graft’s stable location [24, 33, 34]. The bends of stent-graft arms, especially with their segment-like structure, may cause flow disturbances which contribute to the implant clotting and an increased resistance on the stent-graft level [24]. Raben et al. noticed that wider bifurcation angles increases area of low flow and recirculation [35], while Yu and Kwon observed that stent-graft’s design parameters such as porosity, pore density, number of strands, and strut angle have also significant influence on blood hemodynamic. They also observed that an ideal stent-graft should have lower porosity, higher pore density, and higher strut angle [36]. Similarly, Stiehm et al. observed that the stent-graft’s strut thickness is one of the most significant design parameter, and an increase in thickness indicates deceleration of blood flow and recirculation zones [37].

This issue is however complicated and further analysis requires ascertaining how WSS is formed in cases of aneurysms with angulations of the neck and iliac arteries, which may reduce flow and cause turbulence. It will be also important to examine cases where implants have a non-symmetrical bifurcation. Moreover, it may be necessary to determine to what extent changes in flow in the arms or bottlenecks below the implant, can affect stability of the stent-graft fixation. Therefore, preparation of precise recommendations for correct location of the stent-graft or arterioplasty below the implant would be an advantage of computer simulations.

## Conclusion

The proposed algorithm of standardization proved its justness and usefulness in estimating the WSS values. Comparison of the stent-grafts’ geometries with reference cylinders, generated on their basis, allowed the analysis of the implants’ spatial configuration. For stent-grafts close to cylindrical shape (shape factor *φ* close to 1) WSS value was about 267 Pa, while for stent-grafts differ from cylindrical shape (shape factor close to 2) WSS value was about 635 Pa. It was noticed that deformation of the stent-graft’s bifurcation part may impair blood flow hemodynamic which led to increase in the WSS values. Furthermore, an appearance of angular bends or tortuosity in stent-grafts may additionally complicate the estimation of pushing force and should be taken into account in further studies to estimate the risk of implantation failure.
